# BMPER Ameliorates Renal Fibrosis by Inhibiting Tubular Dedifferentiation and Fibroblast Activation

**DOI:** 10.3389/fcell.2021.608396

**Published:** 2021-02-11

**Authors:** Ting Xie, Zunen Xia, Wei Wang, Xiangjun Zhou, Changgeng Xu

**Affiliations:** ^1^Department of Woman's Health Care, Maternal and Child Health Hospital of Hubei Province, Wuhan, China; ^2^Department of Clinical Laboratory, Renmin Hospital of Wuhan University, Wuhan, China; ^3^Department of Urology, The First Affiliated Hospital of Anhui Medical University, Hefei, China; ^4^Institute of Urology, Anhui Medical University, Hefei, China; ^5^Department of Urology, Renmin Hospital of Wuhan University, Wuhan, China; ^6^Department of Urology, The Central Hospital of Wuhan, Tongji Medical College, Huazhong University of Science and Technology, Wuhan, China

**Keywords:** tubulointerstitial fibrosis, BMPER, tubular dedifferentiation, fibroblast activation, signal transduction

## Abstract

Tubulointerstitial fibrosis is both a pathological manifestation of chronic kidney disease and a driving force for the progression of kidney disease. A previous study has shown that bone morphogenetic protein-binding endothelial cell precursor-derived regulator (BMPER) is involved in lung fibrogenesis. However, the role of BMPER in renal fibrosis remains unknown. In the present study, the expression of BMPER was examined by real-time PCR, Western blot and immunohistochemical staining. The *in vitro* effects of BMPER on tubular dedifferentiation and fibroblast activation were analyzed in cultured HK-2 and NRK-49F cells. The *in vivo* effects of BMPER were dissected in unilateral ureteral obstruction (UUO) mice by delivery of BMPER gene via systemic administration of plasmid vector. We reported that the expression of BMPER decreased in the kidneys of UUO mice and HK-2 cells. TGF-β1 increased inhibitor of differentiation-1 (Id-1) and induced epithelial mesenchymal transition in HK-2 cells, and knockdown of BMPER aggravated Id-1 up-regulation, E-cadherin loss, and tubular dedifferentiation. On the contrary, exogenous BMPER inhibited Id-1 up-regulation, prevented E-cadherin loss and tubular dedifferentiation after TGF-β1 exposure. In addition, exogenous BMPER suppressed fibroblast activation by hindering Erk1/2 phosphorylation. Knockdown of low-density lipoprotein receptor-related protein 1 abolished the inhibitory effect of BMPER on Erk1/2 phosphorylation and fibroblast activation. Moreover, delivery of BMPER gene improved renal tubular damage and interstitial fibrosis in UUO mice. Therefore, BMPER inhibits TGF-β1-induced tubular dedifferentiation and fibroblast activation and may hold therapeutic potential for tubulointerstitial fibrosis.

## Background

Chronic kidney disease (CKD), unlike acute kidney injury, is manifested by a gradual decline in kidney function. The shared feature of CKD is tubulointerstitial fibrosis and glomerulosclerosis in tubular and glomerulus compartments (Ruiz-Ortega et al., 2020). Studies have shown that, compared with glomerulosclerosis, the degree of tubulointerstitial fibrosis can better reflect the impaired renal function (Boor and Floege, 2012). Tubulointerstitial fibrosis is not only a pathological appearance of CKD, but also a driving force for the kidney disease progression (Herrera et al., 2018). Hence, strategies for ameliorating renal interstitial fibrosis could blunt kidney disease progression and improve kidney function. Although a variety of molecules have been found to regulate renal fibrosis in basic research, they have not been clinically used. Therefore, it is imperative to develop more effective treatment approaches for renal fibrosis.

Renal tubular cells are easily damaged and only have a certain ability to repair and regenerate. Persistent and repeated injury leads to maladaptive tubular repair and fibrosis, which destroy normal tissue structures (Qi and Yang, 2018). Incompletely epithelial cells repair lead to epithelial dedifferentiation, epithelial mesenchymal transition, and pro-fibrotic factors secretion, which induce fibroblast activation and proliferation (Gewin, 2018).

Renal fibroblasts are located in the renal interstitium and responsible for the production and degradation of extracellular matrix (ECM), maintaining physiological homeostasis and tubule repair after acute kidney injury. Under chronic kidney injury conditions, fibroblasts can be activated by pro-fibrotic cytokines and synthesize and secrete excessive ECM. Therefore, tubular maladaptive repair and fibroblast activation are two key processes in tubulointerstitial fibrosis, apart from tubulointerstitial inflammation. Simultaneous inhibition of tubular dedifferentiation and fibroblast activation may yield better anti-fibrosis effects. However, there is no such research yet.

In search for essential proteins for endothelial precursor cell differentiation, investigators discovered and identified bone morphogenetic protein-binding endothelial cell precursor-derived regulator (BMPER) (Moser et al., 2003). BMPER is recognized as a modulator for BMP signaling and its effect depends on BMP concentration (Kelley et al., 2009). Moreover, BMPER is a multifunctional molecule and implicated in blood vessel development, hematopoietic stem cells (HSC) maturation, endothelial barrier function and inflammation (Moser and Patterson, 2005; Helbing et al., 2017; Lockyer et al., 2017; McGarvey et al., 2017). Notably, BMPER promotes epithelial-mesenchymal transdifferentiation for heart cushions and participates in fibroblast activation and pulmonary fibrosis, indicating its function in fibrogenesis (Dyer et al., 2015; Huan et al., 2015). Our previous study has shown that BMPER expression decreased significantly in hydronephrotic kidneys (Yao et al., 2011). However, it is not clear whether BMPER is an active player in renal fibrosis.

In this study, by using *in vivo* unilateral ureteral obstruction (UUO) and *in vitro* TGF-β1-induced renal fibrosis model, we found that BMPER regulated both tubular dedifferentiation and fibroblast activation, thereby affecting the process of renal fibrosis.

## Materials and Methods

### Materials and Reagents

Recombinant mouse BMPER (2299-CV-050) and recombinant human TGF-β1 (240-B-010) were from R&D Systems (Minneapolis, MN, USA). The primary antibody sources were as follows: anti-collagen I (NB600-408) from NovusBio (CO, USA); anti-BMPER (ab75183), anti-Id-1 (ab230679), anti-fibronectin (ab2413) were from Abcam (Cambridge, UK); anti-α-smooth muscle actin (α-SMA) (ab124964) (BM0002), were from Abcam (Cambridge, UK) and Boshide (Wuhan, China), respectively; anti-E-cadherin (#3195) (20847-1-Ap), were from CST (Cell Signaling Technology, Beverly, MA) and Sanyin (Wuhan, China), respectively; anti-Erk1/2 (#4695), anti-p-Erk1/2 (#4370) from CST (Cell Signaling Technology, Beverly, MA); anti-β-actin (TDY051) from Tiandeyue (Beijing, China). HRP-goat anti-rabbit secondary antibody (AS-1107), CY3-labeled goat anti-mouse secondary antibody (AS-1111) and CY3-labeled goat anti-rabbit secondary antibody (AS-1109) were obtained from ASPEN (Wuhan, China). The BMPER expression plasmid was purchased from OriGene (MR210035) in which BMPER cDNA (NM_028472) was under control of a CMV6 promoter (pCMV6-BMPER). The empty expression plasmid vector pcDNA3 was from Invitrogen (San Diego, CA, USA).

### Animals and Hydronephrotic Kidney Model

The Experimental Animal Center of Wuhan University provided Male C57BL6 mice (body weight 18–20 g). Animals were group-housed in cages in the specific pathogen free animal room. The Institutional Animal Care and Use Committee at The Central Hospital of Wuhan approved and supervised the usage of mice. UUO and sham operation procedures were described previously (Chevalier et al., 2009). Briefly, mice were anesthetized with pentobarbital sodium (50 mg/kg) via intraperitoneal injection. The UUO model was constructed by ligating the left ureter, and the sham operation model was performed with the same surgical procedures without ureter ligation. To investigate the kidney localization and expression changes for BMPER, the mice were allocated into three groups with six mice in each group: (1) mice with sham-operation, (2) mice with 7-day UUO, (3) mice with 14-day UUO. To evaluate the therapeutic effect of exogenous BMPER on renal interstitial fibrosis, the mice were divided into three groups with six mice in each group: (1) mice with sham-operation, (2) seven-day UUO mice treated with empty expression plasmid pcDNA3, and (3) seven-day UUO mice treated with pCMV6-BMPER plasmid. A large number of plasmids were rapidly injected into the mouse circulation through the tail vein, as previously described (Yang et al., 2001a). In brief, 20 μg of plasmid DNA was added to 1.6 ml of saline and injected into mice through the tail vein within 8–10 s. Mice were injected with pCMV6-BMPER plasmid 1 day before operation and 3 days after operation. Control UUO mice were injected with 20 μg of empty vector pcDNA3 plasmid at the same time points in the same manner. All the mice were euthanized 7 or 14 days after UUO or sham operation. The cortex from half of the kidney was dissected and stored for mRNA and protein analysis, and the other half of the kidney was fixed in 4% paraformaldehyde solution for histological and immunohistochemical examination.

### Histological and Immunohistochemical Examination

Fixed kidney tissue was processed through a graded alcohol series, embedded in paraffin wax, and sectioned at 5 μm. Picrosirius red (PSR) staining and hematoxylin and eosin (H&E) staining were operated based on described protocols (Sorensen et al., 2011). H&E staining was used to display tubular atrophy and interstitial expansion. The atrophic tubules were identified and appraised by a scoring system based on the percentage of atrophic tubules (0; 1, <25%; 2, 25–50%; 3, 50–75%; 4, >75%) (Zheng et al., 2019). PSR staining was used to evaluated collagen content. To assess the tubular atrophy and interstitial fibrosis, 20 microscopic fields under high power magnification (400×) were randomly selected. The ratio of the positive red area to the entire area was the percentage of fibrosis, which was calculated with Image J software (National Institute of Health, Bethesda, MD). IHC Tool Box plugin was used for PSR quantification. For immunohistochemistry, the kidney slides were incubated at 4°C overnight with anti-collagen I antibody (1:100), anti-BMPER (1:100), anti-fibronectin antibody (1:100), anti-α-SMA antibody (1:200), and anti-E-cadherin antibody (1:100). The slides were then incubated with secondary antibody (1:5,000) and diaminobenzidine substrate. Finally, nuclei were counterstained with hematoxylin.

### Real-Time Polymerase Chain Reaction (PCR)

Total RNA was extracted from frozen tissue or cultured cells using TRIzol (Invitrogen, Carlsbad, CA, USA). Reverse transcription was performed with FirstStrandcDNA synthesis kit (Thermo scientific, #K1621). Amplified cDNA was used as a template for PCR. Primers were synthesized from Sangon Biological Engineering Technology and Services (Shanghai, China), and specific primers were as follows: Forward 5′-AGGACAGTGCTGCCCCAAATG-3′ and Reverse 5′-TACTGACACGTCCCCTGAAAG-3′ for human BMPER; Forward 5′-GGTGAAGGTCGGTGTGAACG-3′ and Reverse 5′-CTCGCTCCTGGAAGATGGTG-3′ for human GAPDH. Forward 5′-GGTGCGCTGTGTTGTTCATT-3′ and Reverse 5′-TTCTCTCACGCACTGTGTCC-3′ for mouse BMPER; Forward 5′-ATCATCTCCGCCCCTTCTG-3′ and Reverse 5′-GGTCATGAGCCCTTCCACAAC-3′ for mouse GAPDH. Forward 5′-ACTGGGACGACATGGAAAAG-3′ and Reverse 5′-CATCTCCAGAGTCCAGCACA-3′ for rat α-SMA. Forward 5′-TGCTGAGTATGTCGTGGAGTCTA-3′ and Reverse 5′-AGTGGGAGTTGCTGTTGAAATC-3′ for rat GAPDH. Specific primers were chosen from previous studies (Helbing et al., 2010; Zhang et al., 2010; Huan et al., 2015). A final reaction volume of 20 μL sample was amplified according to the manufacturer's protocol (Takara Biotechnology, Japan). Real-time quantifications were carried out on the Prism 7500 SDS (Applied Biosystems, Thermo Fisher Scientific). The relative difference in mRNA expression between groups was calculated with the ΔΔCt method.

### Cell Culture and Treatment

HK-2 and NRK-49F cells were purchased from the American Type Culture Collection and were grown at 37°C in Dulbecco's Modified Eagle's Medium (DMEM) with F12 (Gibco/Life Technologies, Grand Island, NY) supplemented with or without 10% fetal bovine serum (FBS) (Gibco/Life Technologies). When cells grew to about 60% confluence, they were used for *in vitro* experiments. To examine the effect of BMPER on epithelial cell dedifferentiation and fibroblast activation, cells were serum starved overnight and treated with designated amount of BMPER and recombinant TGF-β1 (10 ng/mL for HK-2 or 2 ng/mL for NRK-49F) for the indicated time.

### Cell Viability

Cell viability was measured using the 3-(4,5-dimethylthiazol-2-yl)-2,5-diphenyltetrazolium bromide (MTT) assay. HK-2 and NRK-49F cells were seeded at 1 × 10^4^ cells/well in 96-well plates and exposed to BMPER at a final concentration of 5, 10, 20, 40, 80 nM. The cells were incubated with MTT solution (0.5 mg/ml) for 4 h at 37°C. After MTT solution discarded, 100 μl DMSO was added to each well. Complete dissolution of formazan by evenly shaking the 96-well plates and the optical density (OD) was measured at an absorbance wavelength of 570 nm.

### Western Blot Analysis

Cells and kidney tissues were harvested and lysed with lysis buffer (Biyuntian, Wuhan, China). After the lysates were centrifuged at 12,000×g at 4°C for 20 min, protein concentration was determined using a BCA kit (Biyuntian, Wuhan, China). A total of 20 μg protein was detached on SDS-PAGE gel (Boshide, Wuhan, China) and transferred onto a nitrocellulose membrane (Merck-Millipore, Billerica, MA, USA). The blots were probed with the following primary antibodies overnight: anti-BMPER (1:500), anti-Id1 (1:1,000), anti-E-cadherin (1:1,000), anti-α-SMA (1:1,000), anti-fibronectin (1:500), anti-collagen I (1:500), anti-Erk1/2 (1:2,000), anti-p-Erk1/2(1:1,000), and anti-β-actin (1:10,000). HRP-goat anti-rabbit secondary antibody (1:10,000) was used to conjugate primary antibodies. An enhanced chemiluminescence detection kit (GE Healthcare, Little Chalfont, UK) was used to visualize the bands.

### Immunofluorescence Staining

HK-2 or NRK-49F cells were seeded on coverslips. Forty-eight hours after stimulated with TGF-β1 and BMPER or TGF-β1 and BMPER siRNA, the cells were fixed with 4% paraformaldehyde and stained with primary antibodies. For E-cadherin immunofluorescence staining, HK-2 cells were incubated with the anti-E-cadherin primary antibody (1:100). Subsequently, cells were incubated with CY3 labeled goat anti-rabbit secondary antibody (1:50). For α-SMA immunofluorescence staining, NRK-49F cells were incubated with anti-α-SMA primary antibody (1:200). Subsequently, cells were incubated with CY3 labeled goat anti-mouse secondary antibody (1:50). The fluorescent intensity of images was analyzed by Image J software in 10 randomly chosen, non-overlapping fields (magnification 400×) under a microscope. Mean = IntDen/Area.

### Small RNA Interference

For silencing experiments, 25 pmol human BMPER siRNA (Cat. #AM16708, 127801, Thermo Fisher Scientific, Waltham, MA) and negative control siRNA (Cat. #1022076, Qiangen) were transfected into HK-2 cells using Lipofectamine 2000 (Invitrogen, Paisley, UK) in DMEM/F12 medium without serum and antibiotics. 20 pmol rat low-density lipoprotein receptor-related protein 1 (LRP1) siRNA (Cat. #4390771, s152487, Thermo Fisher Scientific, Waltham, MA) and negative control siRNA (Cat. #4390843 Thermo Fisher Scientific, Waltham, MA) were transfected into NRK-49F cells. After a 6-h transfection, the cells were supplemented with normal medium and cultured overnight, and then exposure to TGF-β1 or BMPER for different times. In our pilot experiment, the treatment concentration used in this study has been optimized.

### Statistical Analysis

All numerical results are presented as means ± SEM. SPSS 18.0 software (SPSS Inc., Chicago, USA) was used for statistical analysis. Each group was compared by ANOVA and Tukey's *post-hoc* analysis. Statistical significance was defined as *P* < 0.05.

## Results

### BMPER Is Down-regulated Under Stress Conditions

**Figure 1:**
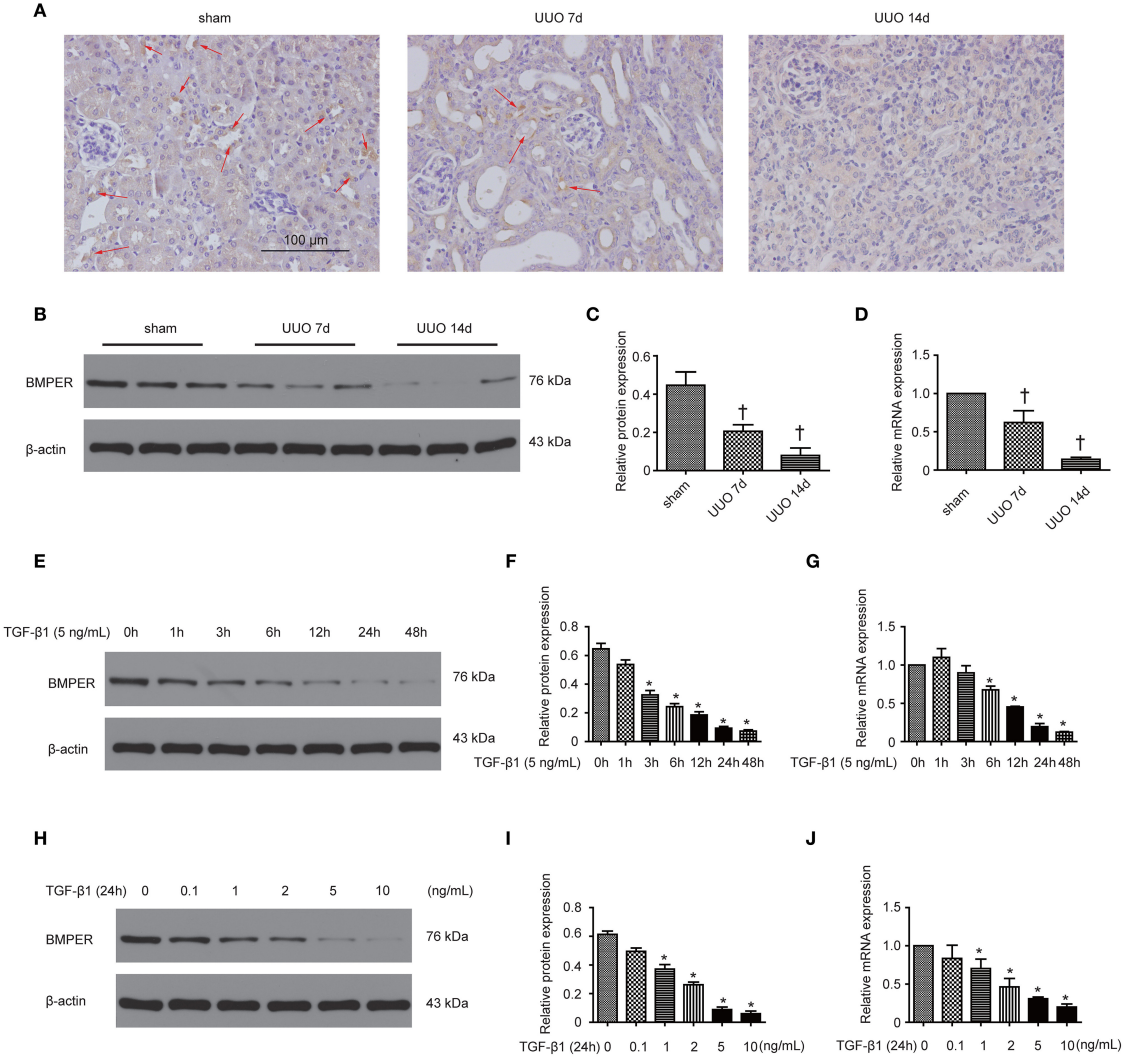
BMPER expression decreased in kidneys after UUO and in HK-2 cells exposed to TGF-β1. Kidneys with indicated UUO time and sham controls were subjected to immunohistochemical staining for BMPER **(A)**. Arrows indicated BMPER-positive tubular cells. Scale bar = 100 μm. BMPER expression in kidneys was measured by Western blot **(B)**. The relative protein expression for BMPER in kidneys was displayed **(C)**. The mRNA expression of BMPER in kidneys with sham operation and UUO was shown **(D)**. Values are the means ± SEM (*n* = 6). HK-2 cells were exposed to TGF-β1 at indicated time and BMPER expression was measured by Western blot **(E)**. The relative protein expression for BMPER in HK-2 cells was displayed **(F)**. The mRNA expression of BMPER in HK-2 cells was shown **(G)**. HK-2 cells were exposed to TGF-β1 at various concentration and BMPER expression was measured by Western blot **(H)**. The relative protein expression for BMPER in HK-2 cells was displayed **(I)**. The mRNA expression of BMPER in HK-2 cells was shown **(J)**. Values are the means ± SEM (*n* = 3). †*p* < 0.05 vs. sham; *p < 0.05 vs. control.

In our previous study, we have shown that BMPER is down-regulated in the hydronephrotic kidneys (Yao et al., 2011). In this study, we examined BMPER expression in UUO mice and HK-2 cells. First, we investigated the localization of BMPER protein in the sham-operated and obstructed kidney. Immunohistochemical staining indicated that BMPER was mainly located in renal tubular epithelial cells in the sham-operated kidneys. However, compared with sham controls, 7-day UUO resulted in decreased BMPER expression. Notably, BMPER was reduced in the dilated and degenerated tubules. Fourteen-day ureteral obstruction further reduced its expression (Figure 1A). This finding was verified by Western blot analysis and RT-qPCR (Figures 1B–D). These observations convincingly suggested that BMPER was related with the degeneration and dedifferentiation of the tubular cells. Then, to further evaluate the changes of BMPER expression *in vitro*, HK-2 cells were treated with TGF-β1. After TGF-β1 treatment, BMPER mRNA and protein expression was also decreased at various time points and different TGF-β1 concentrations (Figures 1E–J). Therefore, BMPER levels by TGF-β1 treatment were dosage- and time-dependent.

### Endogenous BMPER Is Involved in Tubular Dedifferentiation

**Figure 2:**
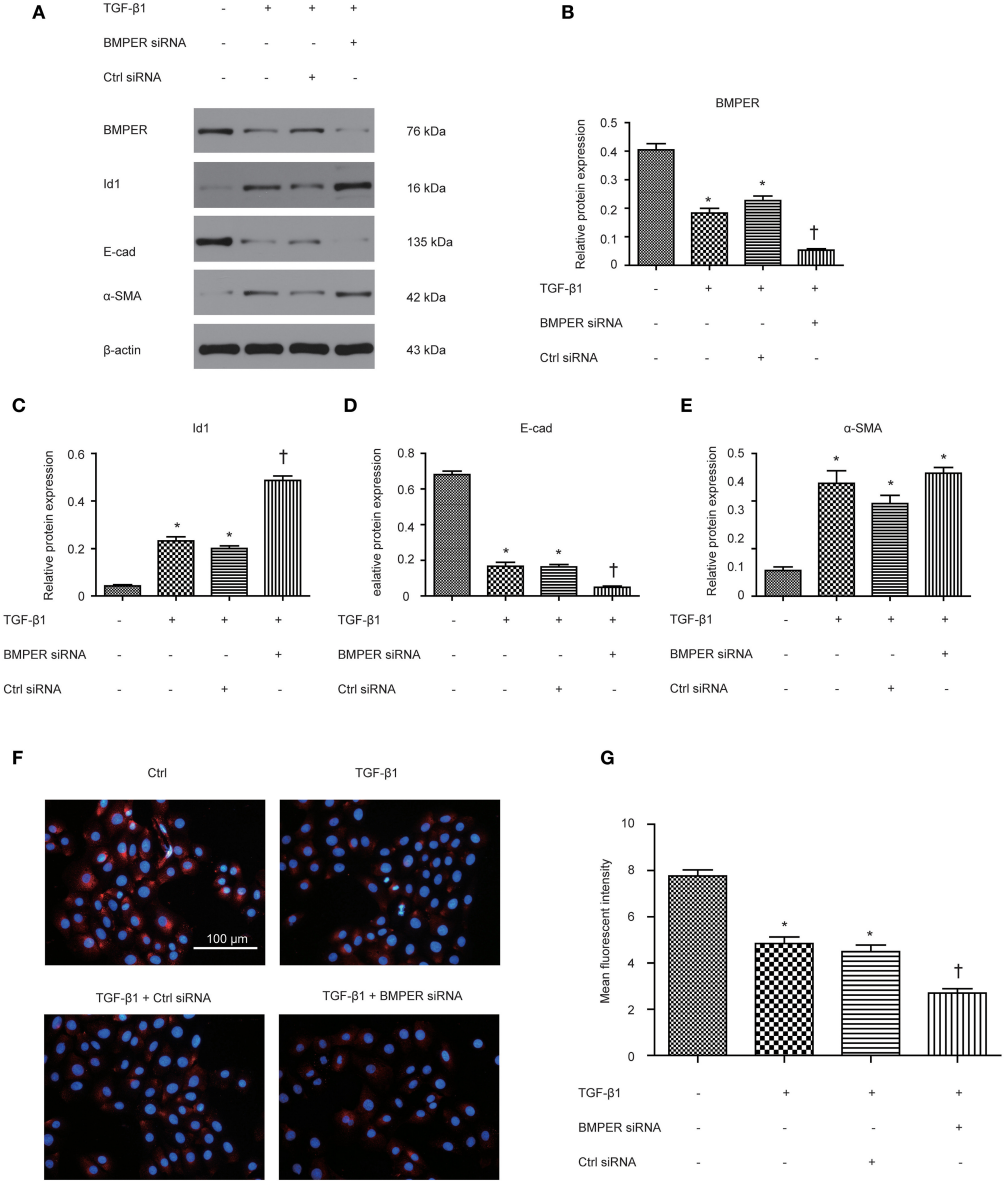
BMPER knockdown aggravated TGF-β1-induced dedifferentiation in HK-2 cells. BMPER, Id1, E-cadherin and α-SMA were measured by Western blot after HK-2 cells with different treatments **(A)**. The relative protein expression for BMPER, Id1, E-cadherin and α-SMA in HK-2 cells was displayed **(C–E)**. Values are the means ± SEM (*n* = 3). Representative photographs of the E-cadherin visualized by indirect immunofluorescence staining in HK-2 cells after various treatments **(F)**. Graphic presentation of mean fluorescent intensity in various groups **(G)**. Scale bar = 100 μm.*p < 0.05 vs. control; †*p* < 0.05 vs. TGF-β1 group.

Epithelial mesenchymal transition (EMT) is an essential process in fibrosis and TGF-β1 plays vital roles in EMT. A previous study has demonstrated that BMPER participates in EMT in heart cushions (Dyer et al., 2015). To assess whether BMPER is involved in EMT mediated by TGF-β1, we examined the E-cadherin and α-SMA levels after BMPER down-regulation with siRNA knockdown in HK-2 cells. As shown in Figures 2A,B,D,E, TGF-β1 treatment led to EMT in HK-2 cells, as evidenced by decreased E-cadherin and increased α-SMA. BMPER knockdown led to decreased E-cadherin expression with TGF-β1 exposure. However, α-SMA remained unchanged after BMPER knockdown and TGF-β1 exposure compared with TGF-β1 exposure alone. Meanwhile, TGF-β1 treatment up-regulated Id1 in HK-2 cells and BMPER knockdown further increased Id1 (Figures 2A,C). Moreover, E-cadherin expression was examined in HK-2 cells by immunofluorescence study. Compared with the control group, tubular cells with BMPER knockdown showed trace E-cadherin staining after TGF-β1 treatment (Figures 2F,G). Therefore, endogenous BMPER was involved in tubular dedifferentiation but not EMT, accompanied by increase in Id1.

### Exogenous BMPER Inhibits Tubular Dedifferentiation

**Figure 3:**
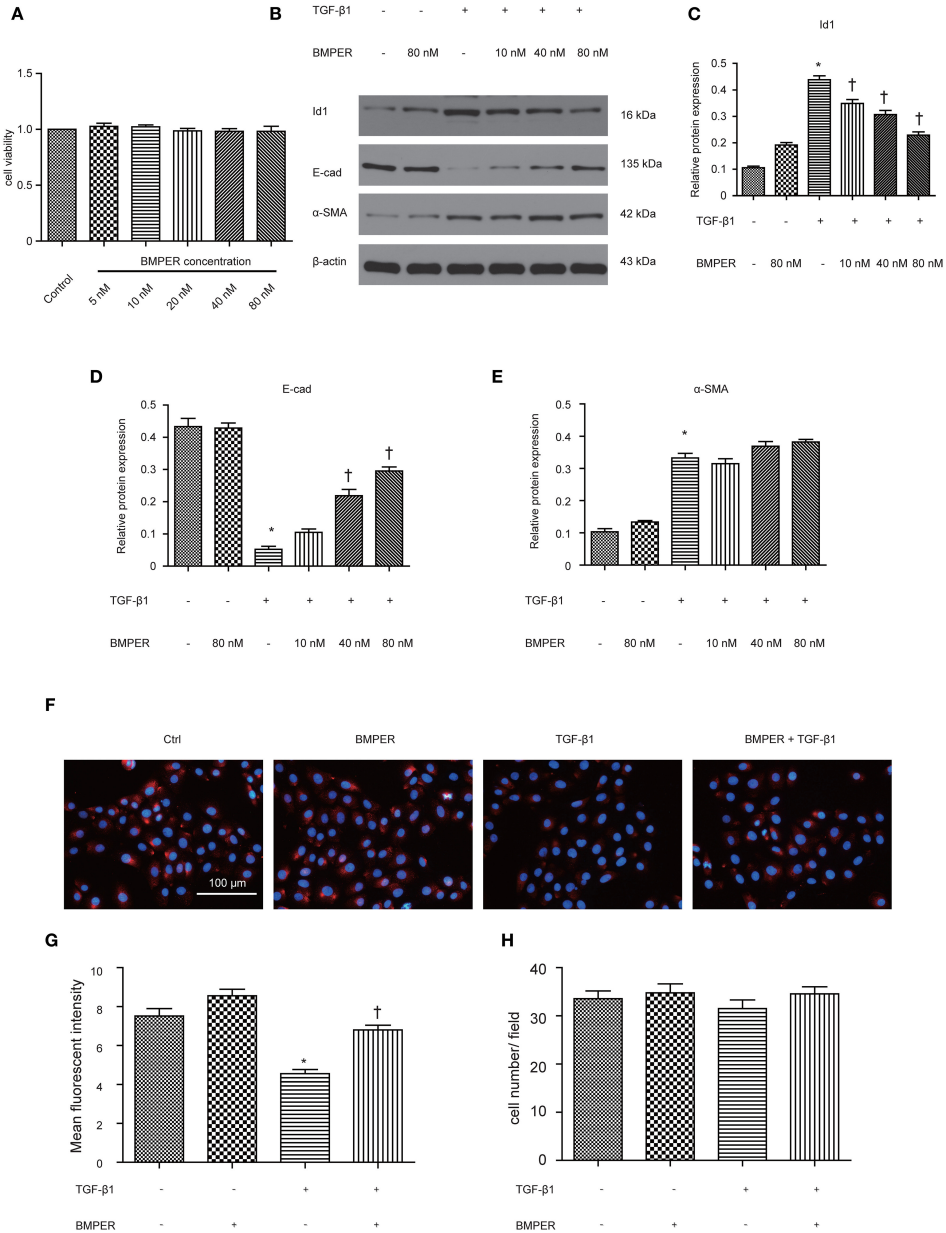
Exogenous BMPER inhibited TGF-β1-induced dedifferentiation in HK-2 cells. HK-2 cells were incubated with increasing amounts of BMPER (5–80 nmol), and cell viability was detected by MTT **(A)**. HK-2 cells were treated with 10 ng/ml TGF-β1 and increasing amounts of BMPER as indicated for 48 h, and the protein expression for Id1, E-cadherin and α-SMA was measured by Western blot **(B)**. The relative protein expression for Id1, E-cadherin and α-SMA in HK-2 cells was displayed **(C–E)**. Values are the means ± SEM (*n* = 3). Representative photographs for E-cadherin in HK-2 cells after various treatments were displayed by immunofluorescence staining **(F)**. Graphic presentation of mean fluorescent intensity in various groups **(G)**. Neither treatment modalities significantly affected HK-2 cell counts **(H)**. Cell numbers were counted after various treatments for 48 h. Scale bar = 100 μm. *p < 0.05 vs. control; †*p* < 0.05 vs. TGF-β1 group.

For investigating the function of BMPER in tubular cells, we studied the phenotypic change after BMPER treatment in HK-2 cells. HK-2 cells were treated with BMPER at various concentrations. Cell viability was examined by MTT assay. BMPER with 5 to 80 nM dose did not affect cell viability (Figure 3A). Therefore, 80 nM of BMPER was used *in vitro* experiments. As shown in Figures 3B–E, BMPER alone did not affect E-cadherin and α-SMA levels. TGF-β1 treatment led to EMT in HK-2 cells. However, BMPER inhibited TGF-β1-meditated E-cadherin loss in a dose-dependent style, without α-SMA change. BMPER alone resulted in a slight increase in Id1, but this change did not achieve statistical difference. BMPER prevented an increase in Id1 induced by TGF-β1 (Figures 3B–E). Immunofluorescence staining for E-cadherin verified this finding (Figures 3F,G). Notably, the difference in E-cadherin expression was not due to the changed cell density after diverse treatments, because any kinds of treatment could not significantly alter HK-2 cell counts (Figure 3H). Therefore, exogenous BMPER inhibited tubular dedifferentiation but not EMT.

### Exogenous BMPER Prevents Fibroblast Activation *via* Inhibiting Erk1/2 Phosphorylation

**Figure 4:**
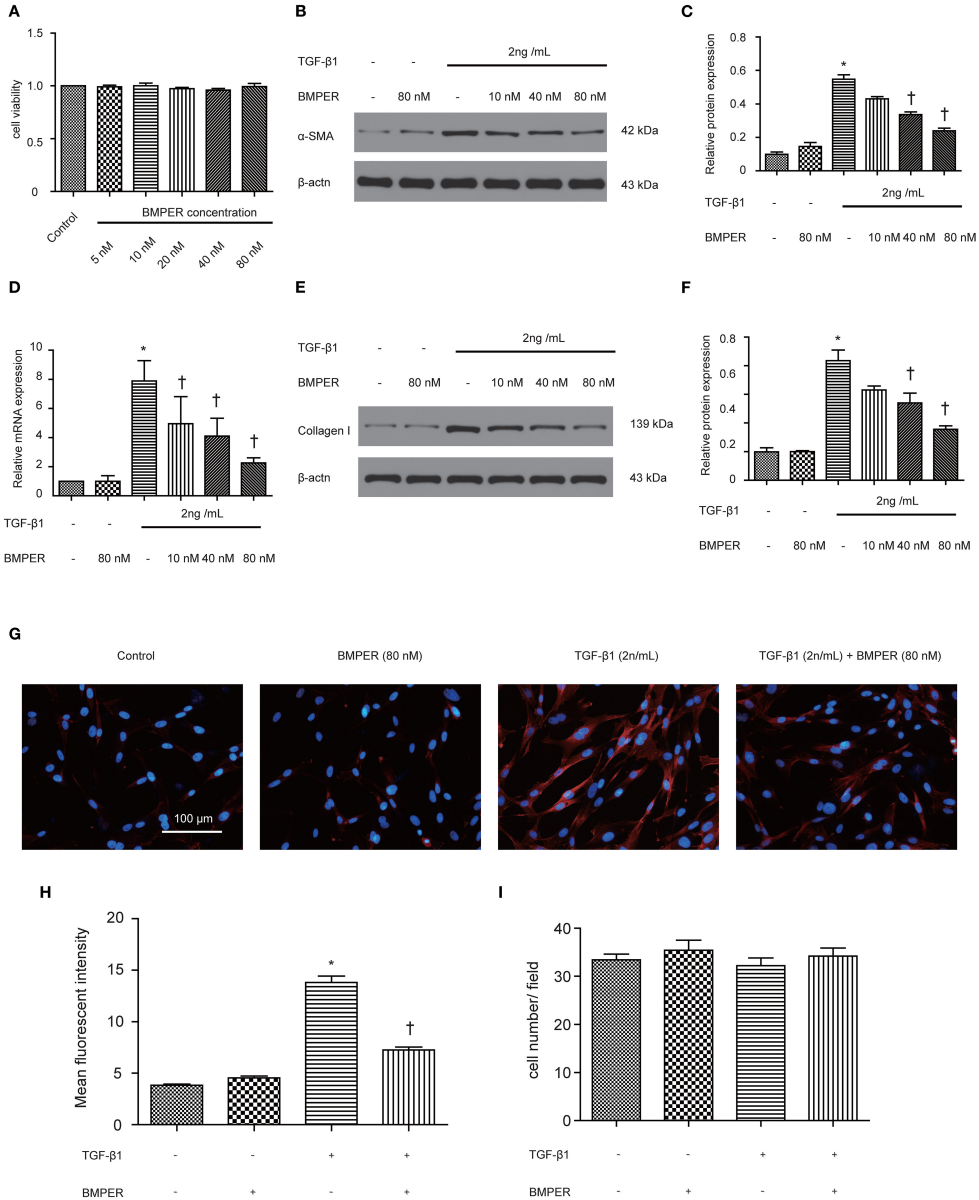
Exogenous BMPER prevented fibroblast activation. NRK-49F cells were incubated with increasing amounts of BMPER (5–80 nmol), and cell viability was detected by MTT **(A)** NRK-49F cells were incubated with a fixed amount of TGF-β1 (2 ng/ml) and increasing amounts of BMPER as indicated for 48 h, and α-SMA was measured by Western blot **(B)**. The relative protein expression for α-SMA in NRK-49F cells was displayed **(C)**. The relative mRNA expression for α-SMA in NRK-49F cells was shown **(D)**. Collangen I was measured by Western blot **(E)** and the relative protein expression for collangen I **(F)**. Values are the means ± SEM (*n* = 3). Representative photographs for α-SMA in NRK-49F cells after various treatments were displayed by immunofluorescence staining **(G)**. Graphic presentation of mean fluorescent intensity in various groups **(H)**. Neither treatment modalities significantly affected NRK-49F counts. Cell numbers were counted after various treatments for 48 h **(I)**. Scale bar = 100 μm. *p < 0.05 vs. control; †*p* < 0.05 vs. TGF-β1 group.

**Figure 5:**
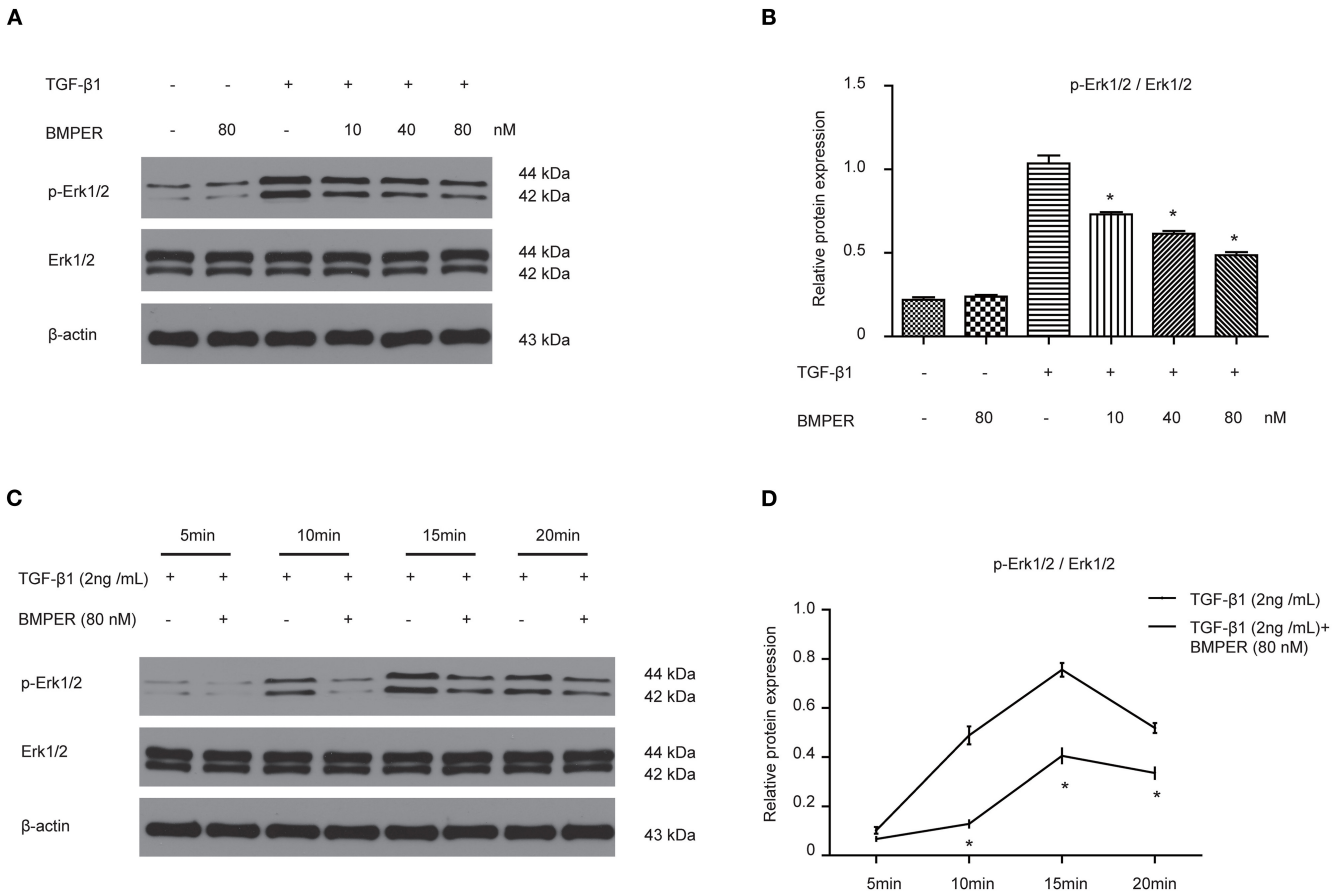
BMPER inhibited Erk1/2 phosphorylation in NRK-49F cells. NRK-49F cells were incubated with 2 ng/ml TGF-β1 and increasing amounts of BMPER as indicated for 20 min, and Erk1/2, p- Erk1/2 were measured by Western blot **(A)**. The relative protein expression for p-Erk1/2 / Erk1/2 in NRK-49F cells was displayed **(B)**. NRK-49F cells were treated with TGF-β1 (2 ng/ml) or TGF-β1 (2 ng/ml) plus BMPER (80 nM) at indicated time, and Erk1/2, p- Erk1/2 were measured by Western blot **(C)**. The relative protein expression for p-Erk1/2 / Erk1/2 in NRK-49F cells was displayed **(D)**. Values are the means ± SEM (*n* = 3). *p < 0.05 vs. TGF-β1 group.

The transition of fibroblast into myofibroblast promotes the development of fibrosis, and various cytokines participate in this process. Blockage of fibroblast activation can attenuate or reverse fibrotic diseases. NRK-49F cells were exposure to BMPER of various concentrations. Cell viability was examined by MTT assay. BMPER with 5 to 80 nM dose did not affect cell viability (Figure 4A). As illustrated in Figure 4B, a dramatic increase in α-SMA in NRK-49F cells after 2 ng/mL TGF-β1 treatment for 24 h. BMPER alone did not change α-SMA expression. However, BMPER could abolish the increase in α-SMA elicited by TGF-β1 treatment (Figures 4B–D). The decreased α-SMA was accompanied by diminished collagen I production (Figures 4E,F). We further examined α-SMA expression in NRK-49F cells via immunofluorescence study. As shown in Figures 4G,H, compared with control groups, BMPER posed an inhibitory effect on up-regulation of α-SMA caused by TGF-β1. Meanwhile, the difference in α-SMA expression was not due to an altered cell number after diverse treatments, because neither treatment modalities significantly affected NRK-49F cell counts (Figure 4I). Therefore, exogenous BMPER could prevent fibroblast activation. Erk1/2 phosphorylation in fibroblast plays an important role in the process of fibrosis. We determined if exogenous BMPER could affect Erk1/2 phosphorylation. As shown in Figures 5A,B, a notable increase in Erk1/2 phosphorylation after a 15 min-TGF-β1 treatment. However, BMPER prevented the Erk1/2 phosphorylation elicited by TGF-β1 in a dose-dependent manner. BMPER alone had no effect on Erk1/2 phosphorylation 15 mins after treatment. BMPER also exhibited time-dependent inhibitory effect on Erk1/2 phosphorylation elicited by TGF-β1 (Figures 5C,D). Together, BMPER prevented fibroblast activation via inhibiting Erk1/2 phosphorylation in fibroblast.

### The Inhibitory Effect of BMPER on Fibroblast Activation Is Dependent of LRP1

**Figure 6:**
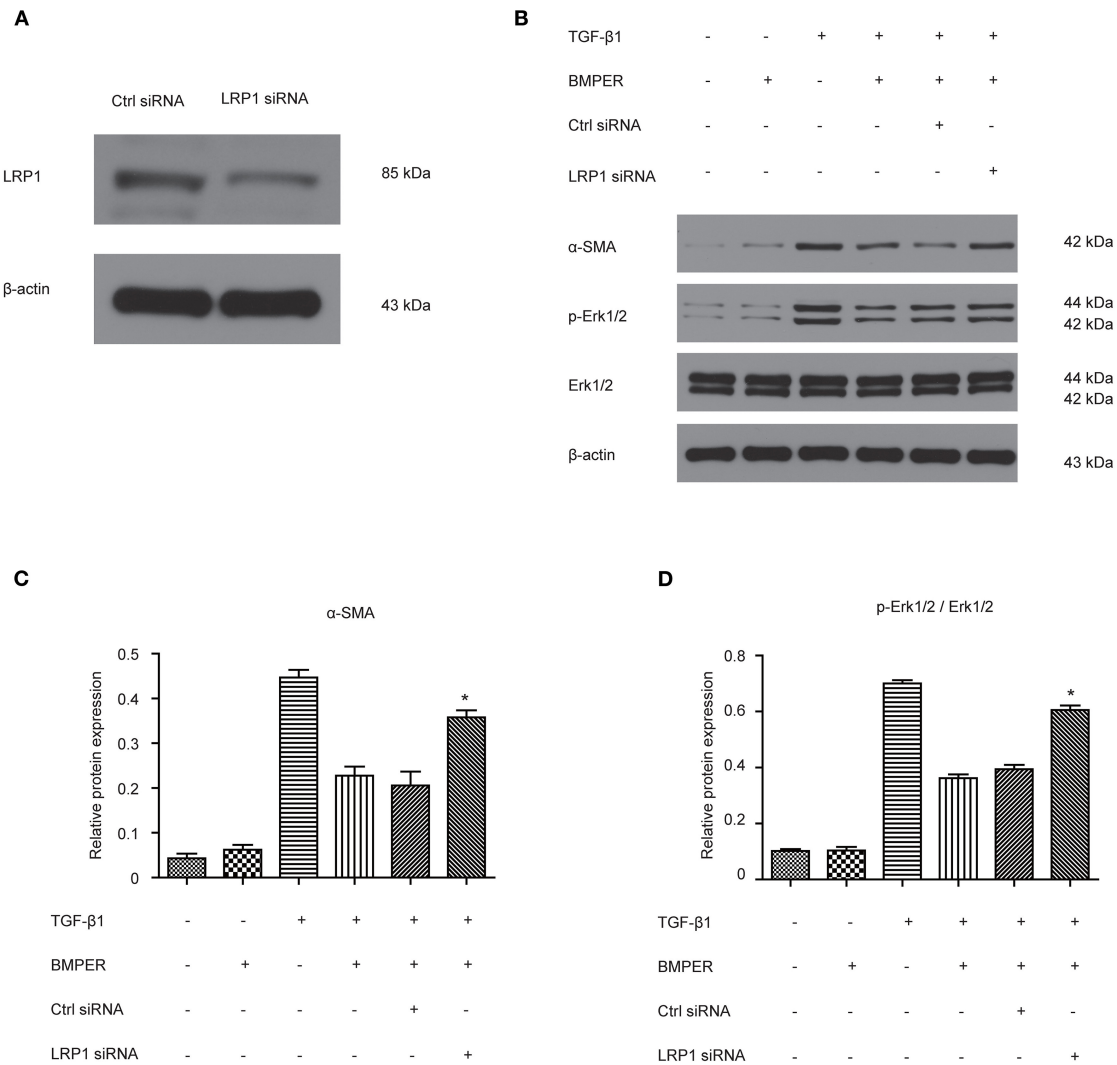
The inhibitory effect of BMPER on Erk1/2 phosphorylation was dependent of LRP1 in NRK-49F cells. The effect of LRP1 siRNA transfection on LRP1 protein expression **(A)** Erk1/2, p- Erk1/2 were measured by Western blot 15 min after NRK-49F cells with different treatments **(B)**. The relative protein expression for α-SMA and p-Erk1/2/Erk1/2 in NRK-49F cells was displayed **(C,D)**. Values are the means ± SEM (*n* = 3). *p < 0.05 vs. cells treated with TGF-β1 plus BMPER.

LRP1 is a cell surface receptor which controls tissue remodeling in several organs (Wujak et al., 2018). LRP1 is also located in fibroblast membrane and engaged in fibrogenesis. We determined if LRP1 was responsible for inhibitory effect of BMPER on fibroblast activation. The result showed LRP1 siRNA transfection led to decreased LRP1 level (Figure 6A). As shown in Figures 6B–D, TGF-β1 elicited an increase in Erk1/2 phosphorylation and α-SMA. However, LRP1 knockdown abrogated the inhibitory effect of BMPER on Erk1/2 phosphorylation and α-SMA. These findings suggested that the inhibitory effect of BMPER on fibroblast activation was dependent of LRP1.

### Exogenous BMPER Attenuates Renal Fibrosis in UUO Mice

**Figure 7:**
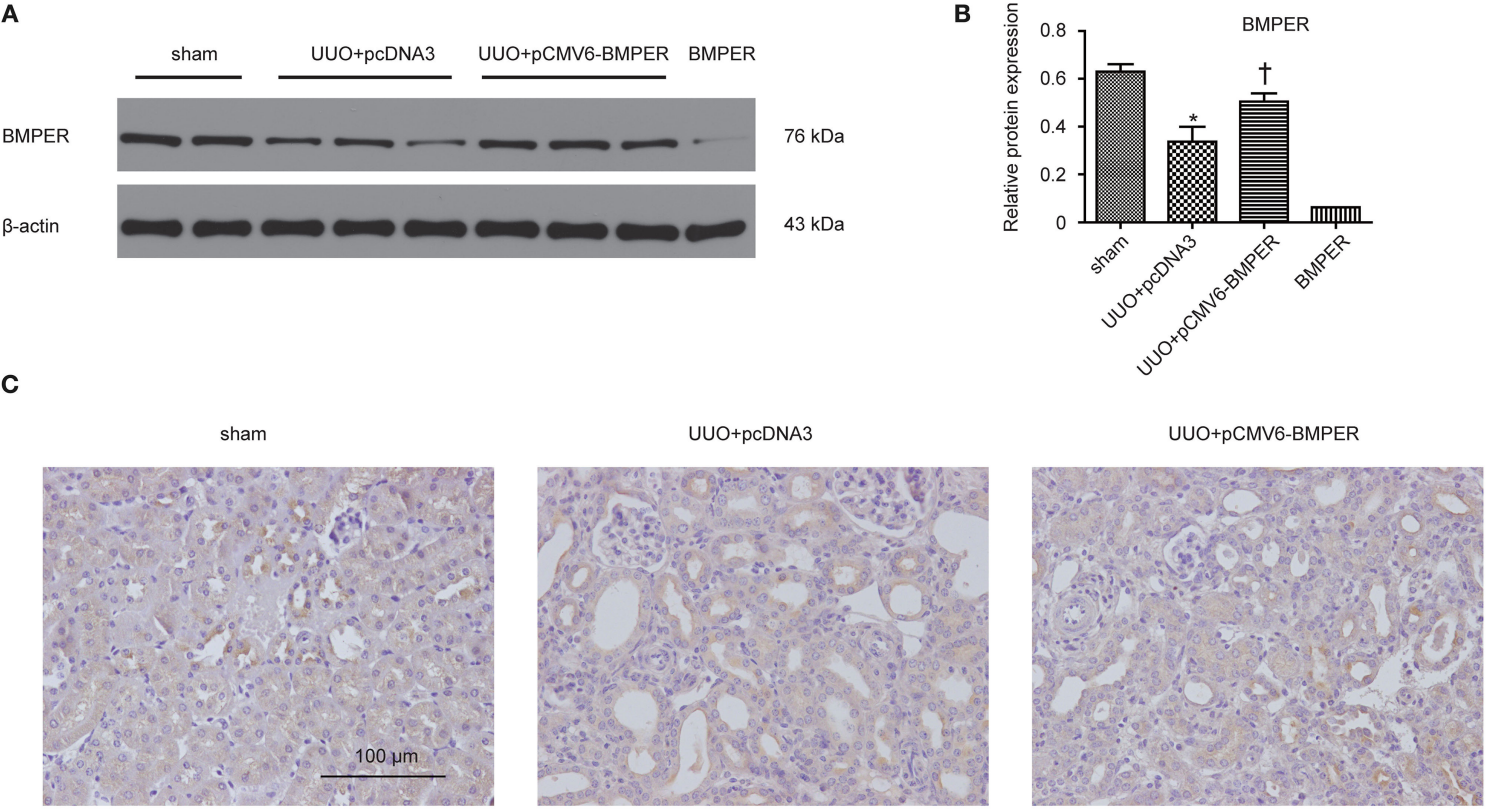
BMPER protein levels increased in the hydronephrotic kidneys after systemic administration of pCMV6-BMPER plasmid. Kidney tissues were homogenized from sham controls and the UUO mice receiving pcDNA3 or pCMV6-BMPER. Western blot demonstrated exogenous BMPER expression in the kidneys. Purified recombinant mouse BMPER (5 ng) was loaded in the last lane **(A)**. The relative protein expression for BMPER in kidneys was displayed **(B)**. Values are the means ± SEM (*n* = 6). Immunohistochemical staining for BMPER were shown **(C)**. Scale bar = 100 μm. *p < 0.05 vs. sham; †*p* < 0.05 vs. UUO+pcDNA3.

**Figure 8:**
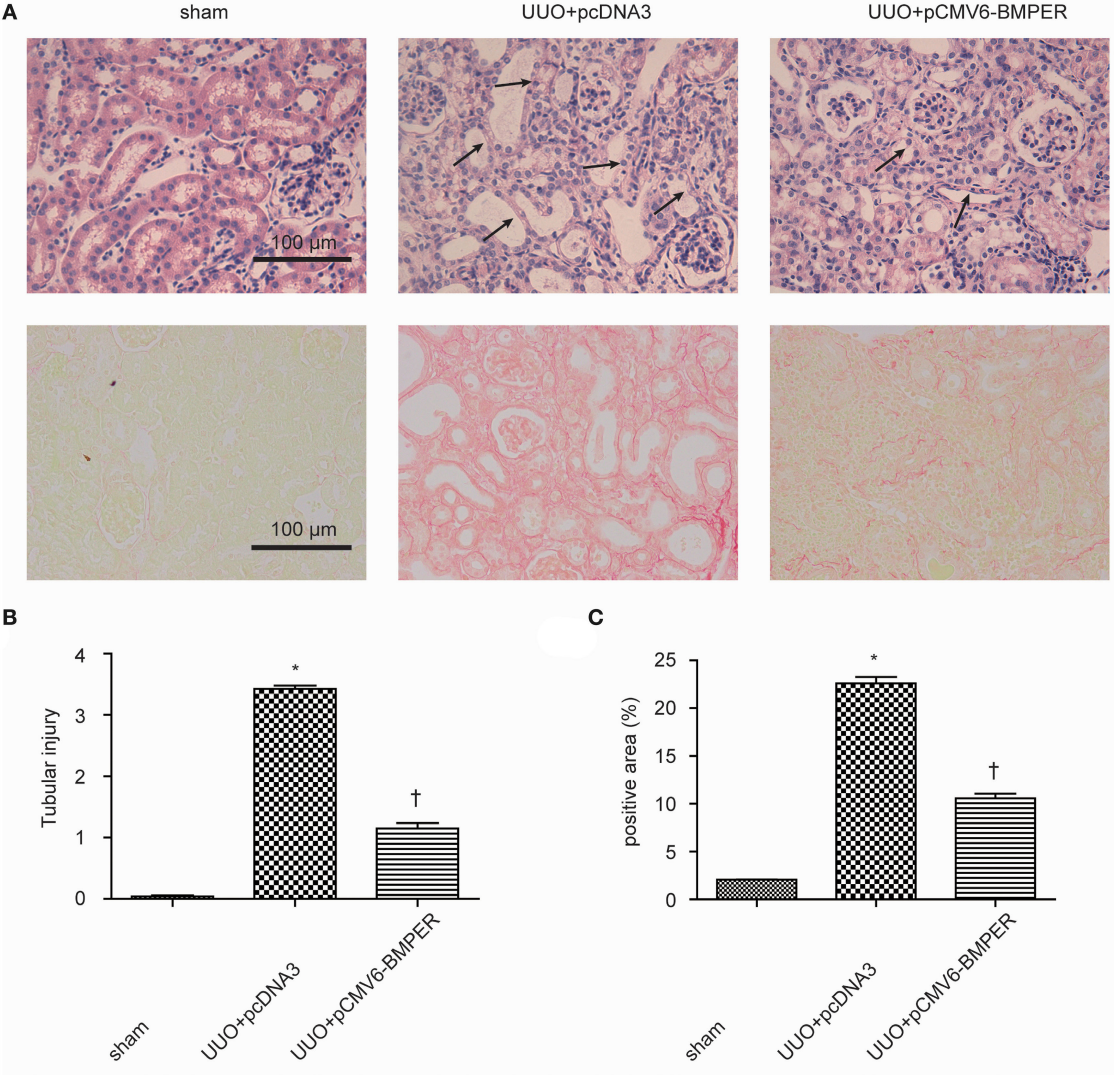
BMPER ameliorated tubule atrophy and interstitial fibrosis. H&E staining showed BMPER ameliorated tubular injury [upper panel of **(A)**]. Arrows indicated tubular atrophy. PSR staining showed BMPER decreased interstitial fibrosis [lower panel of **(A)**]. Semi-quantitative analysis for tubule atrophy and interstitial fibrosis were shown in **(B,C)**, respectively. Scale bar = 100 μm. Values are the means ± SEM (*n* = 6). *p < 0.05 vs. sham; †*p* < 0.05 vs. UUO+pcDNA3.

**Figure 9:**
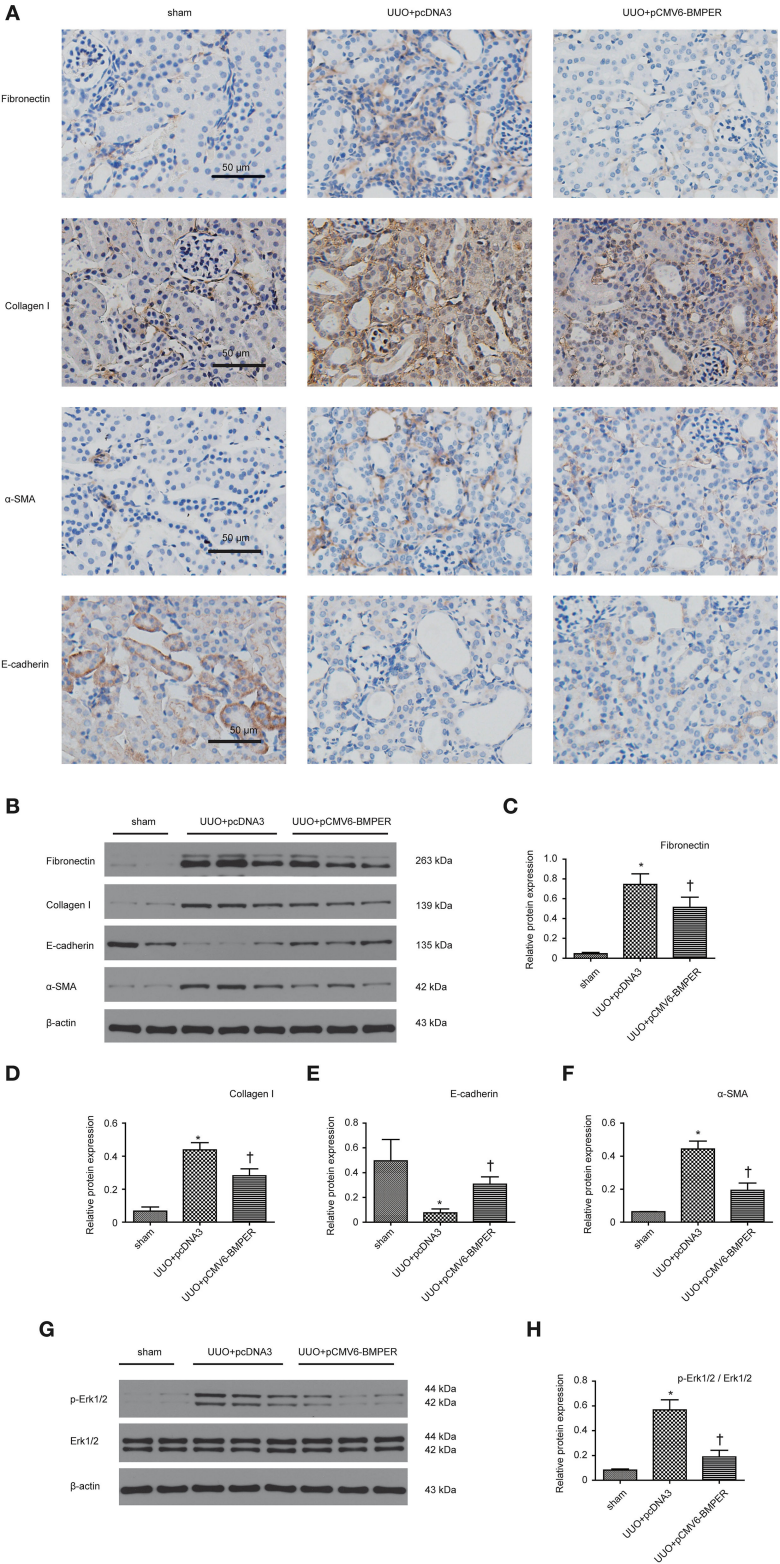
BMPER changed levels of fibrosis-related indicators. Immunohistochemical staining for fibronectin, collagen I, E-cadherin and α-SMA in kidneys was displayed **(A)**. Protein expression for fibronectin, collagen I, E-cadherin and α-SMA in kidneys was measured by Western blot **(B)**. The relative protein expression for fibronectin, collagen I, E-cadherin and α-SMA in kidneys was displayed **(C–F)**. Erk1/2 and p- Erk1/2 were measured by Western blot **(G)**. The relative protein expression for p-Erk1/2 / Erk1/2 in kidneys was displayed **(H)**. Scale bar = 50 μm. Values are the means ± SEM (*n* = 6). *p < 0.05 vs. sham; †*p* < 0.05 vs. UUO+pcDNA3.

In view of the inhibitory effects of BMPER on tubular dedifferentiation and fibroblast activation, two essential processes in renal fibrosis, we determined if BMPER could attenuates renal fibrosis *in vivo*. To deliver exogenous BMPER to the injured kidney, we injected plasmid encoding mouse BMPER cDNA by tail vein using a hydrodynamic gene transfer technique, as previously described (Yang et al., 2001b). Compared with sham-operated mice, UUO induced decrease in BMPER expression. However, BMPER gene transfer restored BMPER level (Figures 7A,B). Immunohistochemical staining showed increased expression of BMPER after hydrodynamic based gene transfer, especially in tubules (Figure 7C). BMPER ameliorated tubule atrophy and interstitial fibrosis, which were assessed by H&E and PSR staining, respectively (Figure 8). Furthermore, immunohistochemical staining displayed that UUO up-regulated the expression of fibronectin, collagen I, and α-SMA and decreased E-cadherin level. However, BMPER corrected these changes (Figure 9A). Western blot analysis corroborated these findings (Figures 9B–F). Meanwhile, UUO promoted Erk1/2 phosphorylation, and BMPER inhibited this process (Figures 9G,H). Collectively, exogenous BMPER attenuated renal fibrosis in UUO mice.

## Discussion

BMPER, as a regulator of BMP signaling, is implicated in blood vessel development, vascular inflammation, HSC maturation and lung fibrosis (Huan et al., 2015; Lockyer et al., 2017; McGarvey et al., 2017; Esser et al., 2018). The findings in the present study demonstrated that BMPER was dramatically down-regulated in both UUO mice and HK-2 cells. BMPER could inhibit tubular dedifferentiation and fibroblast activation. In addition, up-regulation of BMPER could ameliorate tubulointerstitial fibrosis in the kidneys of UUO mice. Therefore, BMPER holds the promise to be a new anti-fibrotic agent.

A previous study has shown that BMPER was decreased in hydronephrotic kidneys from human specimen with serious interstitial fibrosis (Yao et al., 2011). This finding promoted us to seek if BMPER is implicated in renal fibrogenesis and, if so, the underlying mechanism. Among kidney cells, tubular cell was one of the main cell types expressing BMPER. Immunohistochemistry staining showed that 7-day UUO resulted in diminished level of BMPER, especially in dilated and degenerative tubules, which indicated alteration in the kidney microenvironment induced by ureteral obstruction affected its level. Therefore, we examined if TGF-β1, a prominent pro-fibrotic cytokine in kidney after UUO, could suppressed the expression of BMPER. As expected, TGF-β1 could down-regulate BMPER in a time- and dosage-dependent style. These findings suggest that BMPER maybe a regulator for fibrogenesis caused by TGF-β1.

BMPER, initially was identified as a regulator of vascular and blood cell development, could promotes epithelial mesenchymal transition for developing heart cushions (Dyer et al., 2015). Epithelial mesenchymal transition poses an essential role in organ development, fibrosis, and tumor metastasis, and has a relatively conservative molecular mechanism in the three processes (Quaggin and Kapus, 2011). Hence, we assumed that BMPER could affect epithelial mesenchymal transition in renal fibrosis induced by UUO mice. TGF-β1- stimulated HK-2 cells showed increased α-SMA and diminished E-cadherin, indicating EMT in HK-2 cells after TGF-β1 exposure. Endogenous BMPER knockdown aggravated E-cadherin loss in TGF-treated HK-2 cells, and exogenous BMPER restored E-cadherin, without changes in α-SMA after TGF-β1 treatment. These findings suggested BMPER could inhibit tubular dedifferentiation, not EMT. These results were verified by E-cadherin immunofluorescence staining. EMT can be divided into several stages (Liu, 2009). Epithelial cell dedifferentiation, a type of sublethal injury, manifested by loss of epithelial junction molecular, may be the basic event for renal tubular cells to undergo mesenchymal transition (Gwon et al., 2020). E-cadherin is an adhesion receptor between epithelium, which plays vital roles in maintaining epithelium differentiation. Loss of E-cadherin will inevitably cause tubular epithelial cells to lose polarity. Matrix metalloproteinase could disrupt E-cadherin. Loss of E-cadherin promotes the mesenchymal gene expression, and the transcriptional repression of epithelial junction molecule in tubular epithelial cells (Zheng et al., 2009). The data in this study strengthen the significance of BMPER in maintenance of epithelial polarity.

Multiple signaling molecules and pathways regulate the dedifferentiation of epithelial cells. For example, Src families regulate dedifferentiation of tubular cell by activating EGFR/PI3K signaling (Zhuang et al., 2012). Epidermal growth factor receptor (EGFR) activation is necessary for cellular dedifferentiation after injury. Dedifferentiation process is characterized by increased expression of vimentin, decreased expression of E-cadherin, and loss of polarity (Hallman et al., 2008). Inhibitor of differentiation-1 (Id-1), a transcriptional inhibitor, drives tubular epithelial cell dedifferentiation (Li et al., 2007). BMPER impairs proliferation, migration, invasion in lung tumor cell by up-regulation of Id1 (Heinke et al., 2012). Therefore, we assumed that BMPER affected the dedifferentiation of epithelial cells through Id1. As expected, TGF-β1 could induce increase in Id1, and endogenous BMPER knockdown further increased Id1 in TGF-β-treated HK-2 cells. Meanwhile, exogenous BMPER could decrease Id1, accompanied by E-cadherin changes in HK-2 cells after TGF-β1 exposure. These findings indicate that BMPER inhibited dedifferentiation of epithelial cells induced by TGF-β1 through Id1. Besides tumor cell and epithelium, Id1 is also a functional regulator in other cells. BMPER regulates endothelial barrier function by antagonizing BMP4-Smad5-Id1 signaling (Helbing et al., 2017). In lung epithelium, BMPER inhibits BMP activity by antagonizing BMP2-Id1 signaling and preventing decrease of E-cadherin mediated by BMP2, therefore, maintaining the integrity of the epithelium (Helbing et al., 2013). All the results indicate the universality of suppressive effect of BMPER on Id1 signals under various cellular environments.

In normal kidneys, a small number of fibroblasts are quiescent. Upon activation by profibrotic cytokines, fibroblasts acquire a myofibroblast phenotype, express α-SMA, and generate large amounts of ECM components. Activated fibroblasts may be derived from various sources through different mechanisms, including fibroblasts and pericytes activation, tubular epithelial cells transdifferentiation, and circulating fibrocytes recruitment (Mack and Yanagita, 2015). Despite different sources, fibroblast activation is the basis for production of excessive extracellular matrix. BMPER mediates lung fibroblast activation *in vitro* and lung fibrosis in mice *in vivo* (Huan et al., 2015). Therefore, we asked if BMPER was involved in renal fibrosis by affecting fibroblast activation. As expected, BMPER could dose-dependently inhibit rat kidney fibroblast activation, which was displayed by immunofluorescence staining and Western blot for α-SMA.

The extracellular regulated kinase 1/2 (Erk1/2) pathway is activated in the process of renal fibrosis and is related to the differentiation and increased number of renal fibroblasts. For example, Erk1/2 activation was identified in α-SMA-positive fibroblasts in kidney biopsy specimen (Masaki et al., 2004). Erk1/2 activation has also been involved in IL-11 and TGF-β1-induced activation of fibroblasts mediated by NADPH oxidase (Bondi et al., 2010; Schafer et al., 2017). Moreover, inhibition of Erk1/2 blunts the expansion of fibroblasts upon renal fibrosis (Andrikopoulos et al., 2019). Given the importance of Erk1/2 in renal fibrogenesis, we explored whether it mediated the inhibitory effect of BMPER on fibroblast activation. Our *in vitro* results demonstrated that BMPER could dose- and time-dependently suppress Erk1/2 activation in NRK-49F. Our *in vivo* findings revealed that BMPER could suppress Erk1/2 activation in the whole kidney lysis. In tubular cells, TGF-β1-treatment resulted in an increased Erk1/2 activation, but BMPER failed to prevent this process (data not shown). Therefore, the inactivation of Erk1/2 was a fundamental signaling events of BMPER effects on renal interstitial cells. These findings are in agreement with previous results. Trametinib, by inhibiting the Erk1/2 pathway, not only hinders TGF-β1-stimulated renal fibroblast activation, but also improves renal fibrosis in UUO mice (Andrikopoulos et al., 2019). Therefore, anti-fibrotic effect of BMPER may be explained, at least in part, via inhibiting Erk1/2 activation.

LRP1 is a ubiquitously expressed cell receptor protein that regulates the physiological and pathological inflammatory responses to control tissue remodeling in multiple organs (Wujak et al., 2018). High amounts of LRP1 are detected in various tissues and organs, such as in the liver, brain, kidney, and lung (Wujak et al., 2018). LRP1 can improve liver fibrosis by modulating hepatic stellate cells proliferation and migration (Kang et al., 2015). LRP1 also mediates anti-apoptotic effect of tissue-type plasminogen activator (tPA), a serine protease known for producing plasmin, in renal fibroblasts and myofibroblasts (Hu et al., 2008). These studies demonstrate diverse physiological functions, and document the involvement of LRP1 in tissue injury and repair. In our study, LRP1 knockdown abrogated the inhibitory effect of BMPER on Erk1/2 phosphorylation and fibroblast activation, which indicated Erk1/2 phosphorylation mediated the inhibitory effect of BMPER on fibroblast activation. LRP1 mediates different effects through multiple downstream molecules, such as HtrA1 and Erk1/2 (Muratoglu et al., 2013). In our study, LRP1 activated Erk1/2 and promoted fibrogenesis. In line with our result, LRP1 modulates hepatic stellate cells proliferation and migration also by activating Erk1/2 (Kang et al., 2015). In contrast, anti-apoptotic effect of tPA in renal myofibroblasts is meditated by deactivated Erk1/2 (Hu et al., 2008). These inconsistent studies suggest that extracellular cues determine the functional status of Erk1/2 downstream of LPR1.

In this study, BMPER significantly inhibited the down-regulation of E-cadherin and fibroblast activation induced by TGF-β1, and such effect was ignorable without TGF-β1 treatment. Given that E-cadherin down-regulation and fibroblast activation only exist under pathological conditions, BMPER could ameliorate renal fibrosis without affecting constitutive TGF-β1 signaling. In addition to improving tubulointerstitial fibrosis, BMPER can also improve tubular atrophy and preserve tubular epithelial morphology. Tubular atrophy can result from tubular EMT and apoptosis. However, BMPER did not directly improve renal tubular apoptosis and EMT caused by ureteral obstruction. Recent studies have shown that fibroblasts and excessively deposited extracellular matrix can cause tubule epithelial atrophy (Buhl et al., 2020), so we speculate that the preservation of tubule integrity by BMPER is due to its anti-fibrotic effect.

In summary, this study demonstrates that BMPER plays an important role in tubular dedifferentiation, fibroblast activation and tubulointerstitial fibrosis. BMPER holds a promise for inhibiting the progression of chronic kidney diseases.

## Data Availability Statement

The original contributions presented in the study are included in the article/supplementary materials, further inquiries can be directed to the corresponding author.

## Ethics Statement

The animal study was reviewed and approved by Institutional Animal Care and Use Committee at The Central Hospital of Wuhan, Tongji Medical College, Huazhong University of Science and Technology.

## Author Contributions

CX and TX conceived the study. TX and ZX did experiments. TX, WW, and XZ analyzed the data. CX, TX, and ZX drafted the manuscript. The content was approved by all authors. All authors read and approved the final manuscript.

## Conflict of Interest

The authors declare that the research was conducted in the absence of any commercial or financial relationships that could be construed as a potential conflict of interest.

## Abbreviations

BMPER: bone morphogenetic protein-binding endothelial cell precursor-derived regulator
UUO: unilateral ureteral obstruction
Id-1: inhibitor of differentiation-1
CKD: chronic kidney disease
ECM: extracellular matrix
HSC: hematopoietic stem cells
H&E: hematoxylin and eosin
PSR: picrosirius red
α-SMA: α- smooth muscle actin
LRP1: low-density lipoprotein receptor-related protein
EMT: epithelial mesenchymal transition
Erk1/2: extracellular regulated kinase 1/2.
